# Integrative Bioinformatic Analysis of *NHX* Genes in *Spinacia oleracea* L.: From Chromosomes to Stress-Responsive Regulation

**DOI:** 10.3390/genes17030329

**Published:** 2026-03-18

**Authors:** Ummahan Öz

**Affiliations:** Department of Plant and Animal Production, Manisa Celal Bayar University, 45600 Manisa, Türkiye; ummahanoz48@gmail.com or ummuhan.oz@cbu.edu.tr

**Keywords:** Na^+^/H^+^ antiporters, spinach genome, abiotic stress tolerance, bioinformatic analysis

## Abstract

Background/Objectives: The *NHX* gene family plays a critical role in regulating ion homeostasis and enhancing plant tolerance to abiotic stresses. This study aimed to comprehensively analyze the structural, phylogenetic, and functional characteristics of the *NHX* gene family in the genome of *Spinacia oleracea* L. Methods: Through bioinformatic approaches, a total of 44 *NHX* genes were identified, and their chromosomal distribution, exon-intron organization, and conserved motifs were thoroughly characterized. Protein-protein interaction network analysis revealed that SoNHX14, SoNHX20, and SoNHX33 act as central regulators, playing key roles in cellular stress response mechanisms. Furthermore, the majority of SoNHX proteins were predicted to localize primarily to the plasma membrane, endoplasmic reticulum, and vacuole. Promoter analyses indicated a widespread presence of cis-acting elements responsive to stresses such as low temperature, drought, and wounding, as well as elements responsive to plant hormones, suggesting a complex and multilayered regulatory mechanism. Results: miRNA target predictions demonstrated that *NHX* genes are extensively regulated at the post-transcriptional level, predominantly by stress-associated miRNA families. Conclusions: These findings support a central role for the *NHX* gene family in abiotic stress adaptation in *S. oleracea* and provide a valuable foundation for future genetic interventions aimed at improving stress tolerance.

## 1. Introduction

Due to global warming, socioeconomic changes, and the increasing risk of food shortages, increasing the production of efficient vegetables such as lettuce, green beans, spinach, and tomatoes using fewer resources such as less water, fertilizers, pesticides, and land area has become crucial for sustainable agricultural systems and food security [1].

*Spinacia oleracea* L., a member of the Amaranthaceae family, is a widely consumed green leafy vegetable around the world owing to its valuable components, including vitamins (A, C, E, and K), minerals, organic acids, and phenolic acids [2]. It is also an important model plant used by researchers due to its photosynthetic efficiency and salt tolerance [3]. Moreover, *S. oleracea* is known for its high tolerance to cold but exhibits sensitivity to heat stress [4].

Plants have evolved diverse adaptive mechanisms, including stress avoidance and tolerance, to cope with abiotic stress conditions, with various molecular and biochemical processes playing key roles in mediating these responses [5]. Under abiotic stress conditions, various processes occur within different cellular compartments of the plant, including stress sensing, transcription, transcript processing, translation, and post-translational protein modifications [6]. The *Na*^+^/*H*^+^ *antiporter* (*NHX*) genes encode membrane-associated transport proteins that are classified within the cation-proton antiporter (CPA) superfamily and contribute to ion homeostasis under stress conditions by mediating the exchange of Na^+^ and H^+^ ions [7]. Molecular studies on *NHX* genes have been conducted in numerous plants, including *Triticum aestivum* L., *Actinidia chinensis* Planch., *Phaseolus vulgaris* L., *Morus atropurpurea* Roxb. cv. Yuesang, *Punica granatum* L., *Solanum lycopersicum* L., *Cucurbita moschata* Duchesne, *Cucurbita maxima* Duchesne, *Cucurbita pepo* L., *Beta vulgaris* L., *Gossypium barbadense* L., *Canavalia rosea* (Sw.) DC., *Chenopodium quinoa* Willd., *Oryza sativa* L., *Vigna mungo* (L.) Hepper, *Capsicum annuum* L., *Amaranthus tricolor* L., *Brassica napus* L., *Brassica rapa* L., *Brassica oleracea* L., *Glycine max* L., *Medicago truncatula* Gaertn., *Solanum tuberosum* L., and many others, highlighting the association of these genes with stress responses [7,8,9,10,11,12,13,14,15,16,17,18,19,20,21,22,23,24,25,26].

Bioinformatics and sequencing technologies enable the structural and functional analysis of plant genomes, thereby facilitating the systematic processing of large datasets and accelerating scientific discoveries [27]. The accessibility of next-generation tools and techniques used in bioinformatic analyses has made it possible to perform processes that are difficult and costly to achieve with traditional methods, such as modeling gene expression levels, determining the intracellular localization of proteins, and obtaining new insights into metabolite dynamics under stress conditions [28]. In *Spinacia oleracea*, various gene families such as *MADS-box*, *YABBY*, *MYB*, *brassinosteroid-signaling kinase* (*BSK*), *Aux/IAA*, *mitochondrial transcription termination factor* (*mTERF*), and *histone deacetylase* genes have been investigated using bioinformatics tools [4,28,29,30,31,32,33,34]; however, a comprehensive study on the *NHX* gene family is still lacking.

The aim of this study is to comprehensively characterize the *NHX* gene family in *S. oleracea* and, using bioinformatics tools, to reveal molecular features such as gene structure, phylogenetic relationships, protein properties, and cis-regulatory elements, thereby providing insights into the potential functional significance of these genes and their possible involvement in plant stress responses.

## 2. Materials and Methods

### 2.1. Identification and Physicochemical Characterization of NHX Genes in Spinacia oleracea

To identify *NHX* genes in *Spinacia oleracea*, the protein sequences of this species were first downloaded from the NCBI (National Center for Biotechnology Information) database. The analysis was then initiated using the BLASTP tool (Protein-Basic Local Alignment Search Tool) in QIAGEN CLC Genomics Workbench 24.0.1. A PFAM domain search was subsequently performed utilizing the Pfam-A V36 database, which had been previously imported into the software. Upon completion of the search, the PFAM ID (PF0099) was entered, and a filtering step was applied. Candidate proteins were retained based on the presence of this domain, which is characteristic of NHX transporters. The resulting data were transferred to Microsoft Excel, where redundant sequences were removed, and potentially relevant *NHX* genes were retained. Based on the final dataset, the protein, CDS, and genome sequences of the identified *NHX* genes were retrieved from the NCBI database and saved into separate files. Each gene was renamed according to its chromosomal location, using the prefix “So” to denote *Spinacia oleracea* (e.g., *SoNHX1*, *SoNHX2*, etc.). After the gene naming was completed, a new table was created, and the data obtained from NCBI were used to fill the table. For physicochemical characterization, the Expasy ProtParam tool was utilized [35].

### 2.2. Phylogenetic Relationships and Chromosomal Positioning of NHX Genes

To investigate the phylogenetic relationships of *SoNHX* genes, amino acid sequences from *S. oleracea* were analyzed using MEGA 11 software [36]. Multiple sequence alignment was conducted with the MUSCLE algorithm embedded in the platform. The aligned data were exported in MEGA format and used to construct a phylogenetic tree based on the Maximum Likelihood method, employing the Jones–Taylor–Thornton (JTT) substitution model with 1000 bootstrap replicates. The resulting tree was saved in Newick format and visualized using the Interactive Tree of Life (iTOL) v6.7.3 tool [37]. Genes were categorized with distinct color codes, and after applying the necessary formatting adjustments, the final tree was downloaded in PNG format and prepared as a figure. Chromosomal positions were determined using data obtained from NCBI, tabulated, and subsequently uploaded to the MG2C (MapGene2Chromosome) v2.1 software [38]. The obtained figure was downloaded after the necessary adjustments.

### 2.3. Gene Structure and Conserved Protein Motif Analysis

Exon-intron structures of *NHX* genes were examined using the Gene Structure Display Server (GSDS 2.0) [39] by aligning genomic sequences with their corresponding coding sequences (CDS). Conserved motifs within NHX proteins were identified using the MEME Suite version 5.5.1 [40]. The analysis was performed in classical mode, setting the maximum number of motifs to 10 and defining the motif width range as 6 to 50 amino acids. The detected motifs were further analyzed using the MAST (Motif Alignment & Search Tool).

### 2.4. Computational Analysis of Protein Subcellular Localization

For the subcellular localization analysis of NHX proteins, the WoLF PSORT tool [41] (https://wolfpsort.hgc.jp/, accessed on 11 May 2025) was initially utilized. The NHX protein sequences were uploaded to the software, and the resulting predictions were exported to a Microsoft Excel file. These data were then uploaded to ClustVis, a web-based platform for visualizing the clustering of multivariate data (BETA) [42], to generate a heat map. The generated heat map was downloaded and saved as a figure.

### 2.5. Homology Modeling and Protein-Protein Interaction Analysis of NHX Proteins

To predict the 3D structures of NHX proteins, homology modeling was carried out using the intensive mode of the Phyre2 (version 2.2) tool [43], which aligns target sequences with known protein templates. Furthermore, protein–protein interaction networks were analyzed using the STRING v11 database [44] to explore possible functional associations.

### 2.6. Cis-Acting Elements Analysis

The 2 kb upstream promoter sequences of *NHX* genes were retrieved from the NCBI database and subjected to cis-regulatory element analysis using the PlantCARE database [45]. The occurrence of each cis-element, along with the identified motifs with known names and functions, was quantified using Microsoft Excel. For comparative visualization, the processed data were imported into ClustVis [42], and a heat map was constructed based on the distribution patterns of the identified elements.

### 2.7. miRNA Target Prediction and Analysis

The coding sequences (CDS) of *NHX* genes were analyzed using the psRNATarget database [46] to predict potential miRNA targets. The predicted miRNAs were exported to Microsoft Excel and subsequently filtered to select plant-specific miRNAs based on data from the miRBase database. Among these, miRNAs specific to *Arabidopsis thaliana*, a well-established model plant species, were selected. A separate table was constructed listing the *SoNHX* genes targeted by these miRNAs. Additionally, miRNAs reported in the literature to be involved in stress responses were identified. Cytoscape 3.10.3 software [47] was used to visualize the interactions between these miRNA families and their target *NHX* genes, with miRNA families and *SoNHX* genes distinctly color-coded.

## 3. Results

### 3.1. Identification and Physicochemical Characterization of NHX Genes in Spinacia oleracea

A total of 44 *NHX* genes were identified in the *S. oleracea* genome, distributed across chromosomes 1 to 6. The predicted NHX proteins ranged in length from 203 amino acids (SoNHX20) to 1226 amino acids (SoNHX9), with molecular weights between 22.52 kDa and 131.79 kDa. The isoelectric point (pI) values ranged from 4.88 to 9.38, with the majority of proteins having pI values below 7, indicating that most SoNHX proteins possess acidic characteristics. The instability index values, which provide a computational estimation of protein stability based on amino acid composition, ranged from 27.56 to 45.19. Based on these values, 35 proteins were classified as stable and 9 as unstable (Supplementary Table S1). The majority of the genes were found to encode structurally stable proteins, suggesting their potential functional importance under physiological conditions.

### 3.2. Phylogenetic Relationships and Chromosomal Positioning of NHX Genes

**Figure 1:**
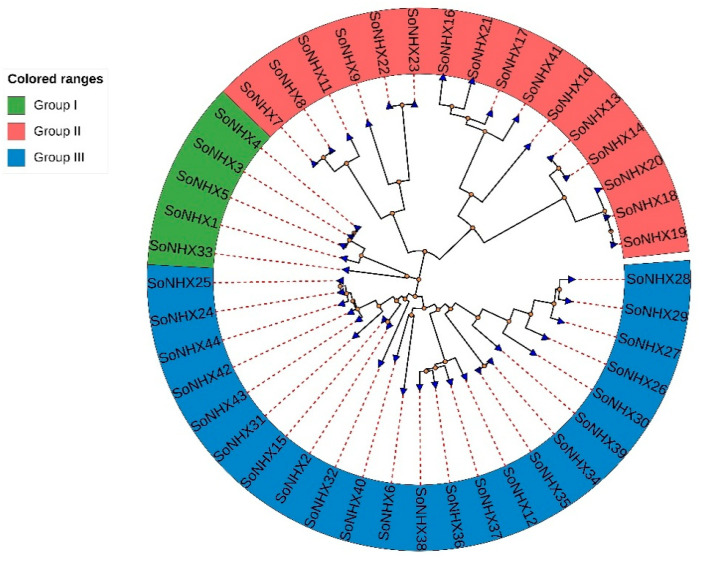
Phylogenetic tree of the *NHX* gene family in *S. oleracea* constructed using the Maximum Likelihood method based on amino acid sequences. Bootstrap analysis with 1000 replicates was performed to assess branch support.

As a result of the phylogenetic analysis, the constructed phylogenetic tree revealed that the *NHX* genes were clustered into three main groups (Figure 1). Group III contained the highest number of genes (23), whereas Group I had the fewest (5). In Group I, *SoNHX1* and *SoNHX33* were clearly separated from the other genes. Group II comprised 16 genes and was distinctly divided into two subclades. Within this group, *SoNHX9*, *SoNHX10*, *SoNHX11*, and *SoNHX17* also showed clearly distinct positions. In Group III, a high degree of branching was observed, suggesting that this group may possess substantial genetic diversity.

**Figure 2:**
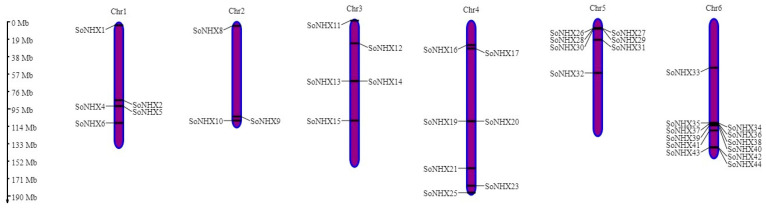
Chromosomal positioning of the *SoNHX* gene family members.

*NHX* genes were mapped across all chromosomes of *S. oleracea*, indicating a genome-wide distribution. Chromosome 6 contained the highest number of *NHX* genes, followed by chromosome 4, while chromosome 2 contained the fewest (Figure 2). Owing to identical start positions, only one representative gene from each of the following gene pairs is depicted in Figure 2: *SoNHX3–SoNHX4*, *SoNHX7–SoNHX8*, *SoNHX18–SoNHX19*, *SoNHX22–SoNHX23*, and *SoNHX24–SoNHX25*.

### 3.3. Gene Structure and Conserved Protein Motif Analysis

**Figure 3:**
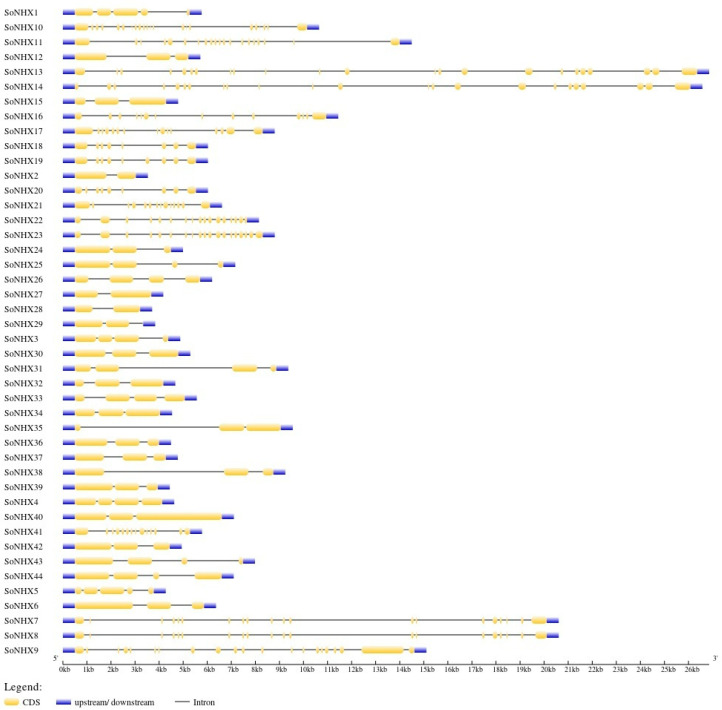
Intron and Exon Distribution Among *SoNHX* Gene Family Members.

Structural analysis of the *SoNHX* gene family revealed notable variation in exon–intron organization among the identified genes. *SoNHX13* and *SoNHX14* contained the highest number of exons and introns, with 23 exons and 22 introns each, and *SoNHX13* was the longest gene. In contrast, *SoNHX2*, *SoNHX27*, and *SoNHX28* comprised only two exons and a single intron, with *SoNHX2* being the shortest gene. Interestingly, *SoNHX29* exhibited a unique structure in which the 3′ region was directly associated with an intron, differing from the typical 5′-exon–intron–exon–3′ arrangement seen in other genes. Additionally, *SoNHX7* and *SoNHX8* featured relatively long intronic regions (Figure 3).

**Figure 4:**
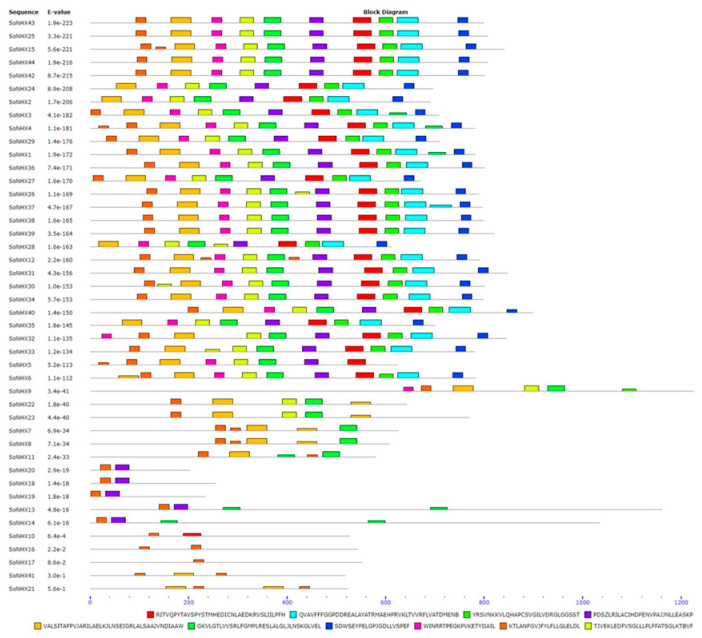
Conserved Motif Organization in SoNHX Proteins. Different colored boxes represent distinct conserved motifs.

As a result of the conserved motif analysis conducted on SoNHX proteins, it was determined that SoNHX1, SoNHX3, SoNHX4, SoNHX6, SoNHX12, SoNHX15, SoNHX25, SoNHX26, SoNHX27, SoNHX28, SoNHX29, SoNHX30, SoNHX31, SoNHX32, SoNHX34, SoNHX36, SoNHX37, SoNHX38, SoNHX39, SoNHX40, SoNHX42, SoNHX43, and SoNHX44 proteins contained all the analyzed motifs. In contrast, SoNHX16 (with two copies of motif 9) and SoNHX17 (only motif 9) were found to contain only a single motif. Additionally, SoNHX10 was found to possess only motifs 1 and 9; SoNHX18, SoNHX19, and SoNHX20 only motifs 4 and 9; and SoNHX21 and SoNHX41 only motifs 5 and 9 (Figure 4).

### 3.4. Computational Analysis of Protein Subcellular Localization

**Figure 5:**
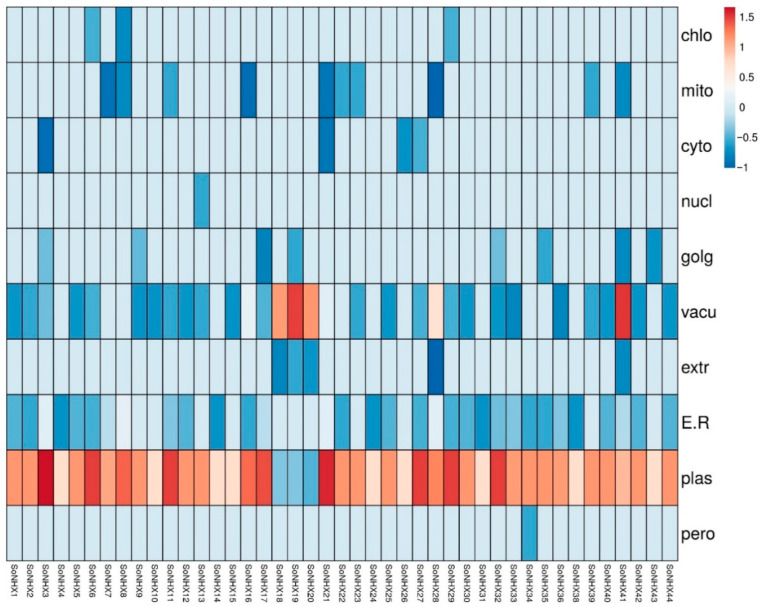
Predicted subcellular localization of SoNHX proteins in *S. oleracea*. The heat map illustrates the predicted localization patterns of SoNHX proteins across different cellular compartments based on computational analysis. Each color intensity represents the relative prediction score for localization in a specific organelle. Abbreviations: chlo, chloroplast; mito, mitochondrion; cyto, cytoplasm; nucl, nucleus; golg, Golgi apparatus; vacu, vacuole; extr, extracellular space; ER, endoplasmic reticulum; plas, plasma membrane; pero, peroxisome.

SoNHX proteins were predicted to localize in various subcellular compartments, including the chloroplast, mitochondria, cytoplasm, nucleus, Golgi apparatus, vacuole, extracellular space, endoplasmic reticulum, plasma membrane, and peroxisome. All SoNHX proteins were found to be localized to the plasma membrane. Among them, SoNHX34 was uniquely localized to the peroxisome, whereas SoNHX13 was exclusively detected in the nucleus. Additionally, SoNHX37 was found to be present only in the plasma membrane. Following the plasma membrane, the most common subcellular localizations of SoNHX proteins were the endoplasmic reticulum (32 proteins) and the vacuole (31 proteins) (Figure 5).

### 3.5. Homology Modeling and Protein-Protein Interaction Analysis of NHX Proteins

The analysis of the secondary structural components of NHX proteins revealed that α-helix structures are generally predominant. In this context, SoNHX18 was identified as the protein with the highest α-helix content at 82%, followed by SoNHX20 (81%) and SoNHX19 (79%). Another notable finding is that SoNHX19 exhibited the highest proportion of transmembrane helices (TM helices), with a rate of 66%. In terms of β-strand structures, SoNHX10, SoNHX16, SoNHX17, SoNHX18, SoNHX19, SoNHX20, and SoNHX41 were found to lack this structural element. However, some proteins contained very low levels of β-strands: 1% in SoNHX8, SoNHX11, and SoNHX21; 2% in SoNHX7 and SoNHX22; 3% in SoNHX23; and 4% in SoNHX9. Furthermore, in terms of disordered regions, SoNHX9 exhibited the highest proportion at 34%, distinguishing it from the other proteins.

**Figure 6:**
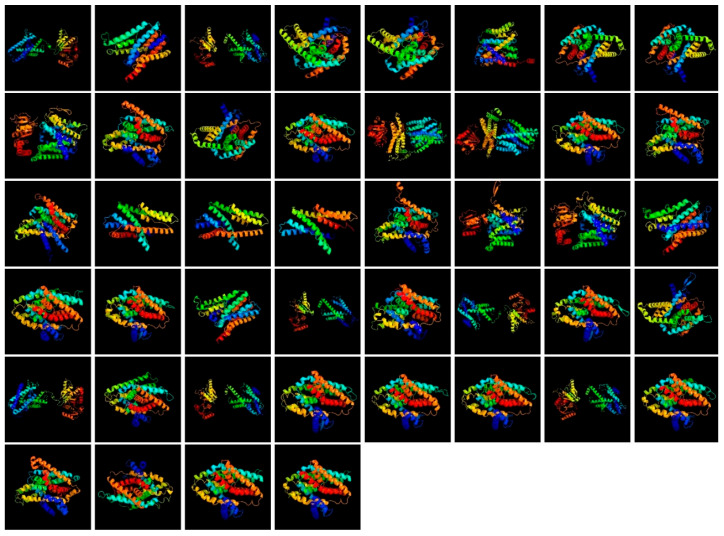
Three-dimensional structural models of SoNHX proteins arranged according to gene number. The models illustrate the overall structural organization of the proteins, including α-helices, β-sheets, and loop regions, and highlight structural differences among SoNHX proteins.

Examination of Figure 6 based on protein homology modeling reveals a general predominance of α-helix structures across the analyzed proteins. However, notable structural variations were observed among SoNHX1, SoNHX3, SoNHX9, SoNHX13, SoNHX14, SoNHX22, SoNHX23, SoNHX28, SoNHX30, SoNHX33, SoNHX35, and SoNHX39. SoNHX1 exhibited two connected domains predominantly composed of α-helices, along with parallel and antiparallel β-sheets, β-turns, and long loops. SoNHX3 featured two distinct regions with α-helices dominating one and β-sheets the other, both containing parallel and antiparallel β-sheets, β-turns, and long loops. Similarly, SoNHX9 consisted of two separate domains, one with α-helices and loops and the other combining parallel β-sheets, α-helices, and loops. SoNHX13 presented three connected domains, with β-sheets predominant in the first and α-helices in the latter two, accompanied by antiparallel β-sheets, β-turns, and long loops. SoNHX14 closely resembled SoNHX13, with three regions showing antiparallel β-sheets in the first and α-helices in the second and third. SoNHX22 had two spatially separated domains: the first rich in parallel β-sheets, α-helices, and long loops, and the second dominated by α-helices with a single antiparallel β-sheet. SoNHX23 showed two connected domains, one containing parallel β-sheets, α-helices, and long loops, and the other only α-helices and long loops. SoNHX28 comprised two distinct domains, with the first containing parallel β-sheets, α-helices, β-turns, and long loops, and the second α-helices and long loops. SoNHX30 also exhibited two connected domains, with the first composed solely of α-helices and long loops and the second including antiparallel β-sheets, β-turns, α-helices, and long loops. SoNHX33 featured two connected domains: the first with α-helices and long loops, and the second including parallel and antiparallel β-sheets, β-turns, α-helices, and long loops. SoNHX35 had two distinct domains, with the first dominated by parallel β-sheets and β-turns alongside α-helices and long loops, and the second consisting only of α-helices and long loops. Finally, SoNHX39 contained two separate domains: the first with parallel and antiparallel β-sheets, β-turns, and long loops, and the second with α-helices, antiparallel β-sheets, and long loops.

**Figure 7:**
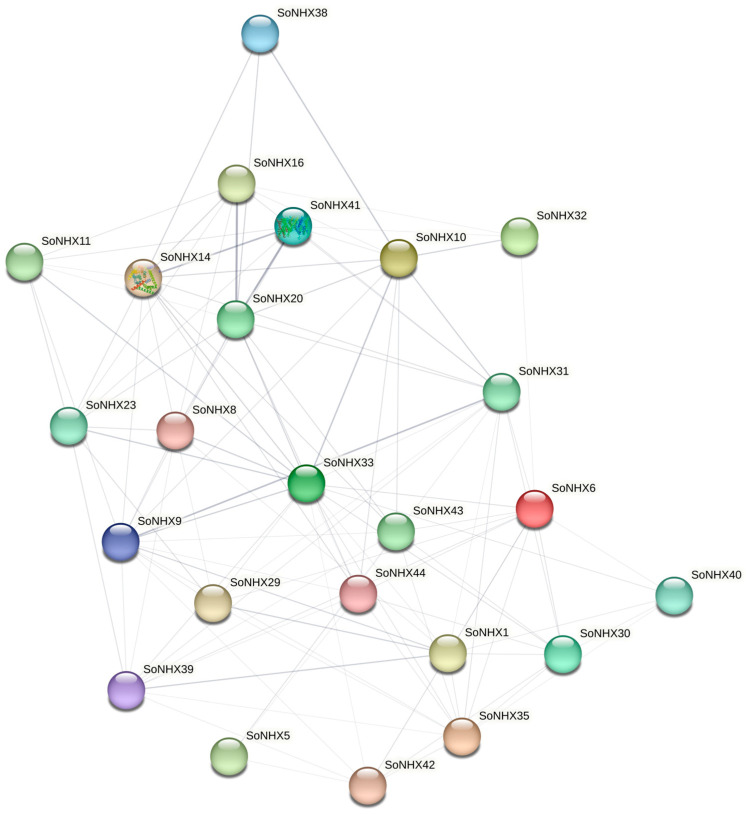
Predicted protein–protein interaction network of SoNHX proteins. Nodes represent SoNHX proteins, and edges indicate predicted functional associations between proteins.

Protein–protein interaction analysis indicates that the SoNHX protein family forms a highly dense and complex network. Within this network, SoNHX14, SoNHX20 and SoNHX33 exhibit the highest number of interactions, suggesting that they occupy central positions and may have regulatory functions. In contrast, members such as SoNHX38 and SoNHX40 display fewer interactions, indicating more peripheral roles. The interaction map highlights both direct and indirect associations among the proteins, implying that SoNHX proteins function in a coordinated manner in cellular processes (Figure 7).

### 3.6. Cis-Acting Elements Analysis

**Figure 8:**
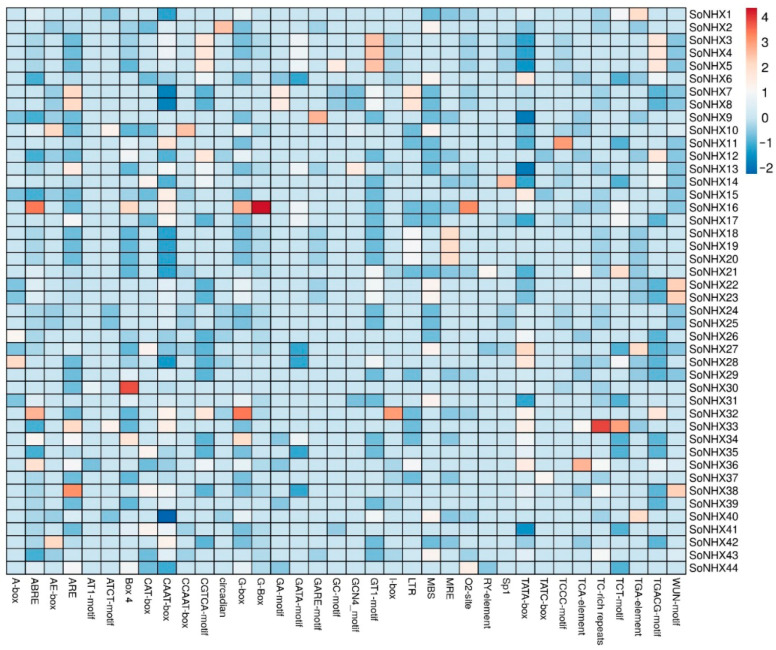
Heat map showing the distribution of cis-acting elements in the promoter regions of *SoNHX* genes.

As a result of the cis-acting element analysis, motifs with known functions were classified in a table, their frequencies were recorded, and a heat map was generated accordingly (Figure 8). The highest number of cis-acting elements was identified in the *SoNHX16* gene (144), followed by *SoNHX32* (142) and *SoNHX33* (135). The lowest number was detected in the *SoNHX9* gene (69). In terms of motif diversity, the *SoNHX21* gene exhibited the highest variety, with 23 different motifs. This was followed by *SoNHX7*, *SoNHX8,* and *SoNHX13*, each containing 22 different motifs. The lowest motif diversity was observed in the *SoNHX30* gene, with 8 different motifs (Supplementary Table S2).

Moreover, several stress-responsive cis-elements were identified in the promoter regions of *SoNHX* genes, such as LTR (responsive to low temperature), MBS (associated with drought stress), TC-rich repeats (involved in defense and stress responses), and WUN-motifs (linked to wounding signals). In addition, numerous motifs associated with light responsiveness were identified. These include the 3-AF1-binding site, 4cl-CMA2b, AAAC-motif, ACA-motif, ACE, AE-box, AT1-motif, ATC-motif, ATCT-motif, Box II, Box 4, chs-CMA1a, chs-CMA2a, G-box, G-Box, GA-motif, Gap-box, GATA-motif, GATT-motif, GT1-motif, GTGGC-motif, I-box, L-box, LAMP-element, MRE, Sp1, TCCC-motif, and TCT-motif (Supplementary Table S2).

Various cis-acting elements responsive to phytohormones were identified in the *SoNHX* genes. These include ABRE (abscisic acid-responsive), AuxRR-core, TGA-element, and TGA-box (auxin-responsive), CGTCA-motif and TGACG-motif (methyl jasmonate-responsive), GARE-motif, P-box, and TATC-box (gibberellin-responsive), as well as SARE and TCA-element (salicylic acid-responsive) motifs. These findings suggest that *SoNHX* genes may play significant roles in various physiological processes and stress responses regulated by hormonal signaling. Additionally, motifs associated with important functions such as anaerobic induction, meristem expression, circadian control, anoxia-specific inducibility, endosperm expression, differentiation of palisade mesophyll cells, regulation of flavonoid biosynthetic genes, cell cycle regulation, and seed-specific regulation were identified in the *SoNHX* genes (Supplementary Table S2).

One of the notable findings from the cis-acting element analysis was that certain motifs were uniquely detected in specific *SoNHX* genes: 4cl-CMA2b only in *SoNHX28*, ACA-motif only in *SoNHX14*, chs-CMA2a only in *SoNHX38*, Gap-box only in *SoNHX1*, SARE only in *SoNHX32*, and TGA-box only in *SoNHX26* (Supplementary Table S2). It was observed that certain cis-elements were more abundant in specific *SoNHX* genes, such as ABRE in *SoNHX16*, ARE in *SoNHX38*, Box 4 in *SoNHX30*, G-box in *SoNHX32*, and TC-rich repeats in *SoNHX33* (Figure 8).

### 3.7. miRNA Target Prediction and Analysis

The data on miRNA interactions targeting *SoNHX* genes are presented in Supplementary Table S3. *SoNHX11* showed the highest number of predicted miRNA-binding interactions (595), followed by *SoNHX33* (558) and *SoNHX9* (496). In contrast, *SoNHX18* exhibited the lowest number of predicted interactions, with 98 potential miRNA-binding events.

**Figure 9:**
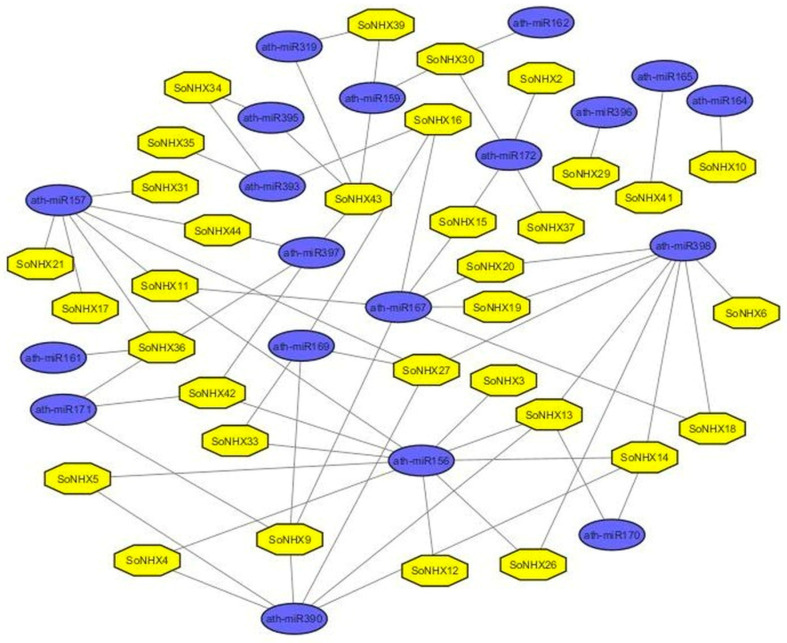
Predicted interaction network between stress-related Arabidopsis thaliana miRNAs and *SoNHX* genes. Circular nodes represent miRNAs and square nodes represent *SoNHX* genes, while edges indicate predicted regulatory interactions.

Among the identified miRNAs, only those belonging to *Arabidopsis thaliana* were selected, and among them, miRNAs known to be associated with stress responses, such as ath-miR156, ath-miR157, ath-miR159, ath-miR161, ath-miR162, ath-miR164, ath-miR165, ath-miR167, ath-miR169, ath-miR170, ath-miR171, ath-miR172, ath-miR319, ath-miR390, ath-miR393, ath-miR395, ath-miR396, ath-miR397 and ath-miR398, were filtered. The interactions between these ath-miRNAs and *SoNHX* genes are presented in Figure 9. Among these, ath-miR156 was identified as the miRNA targeting the highest number of genes, including *SoNHX3*, *SoNHX4*, *SoNHX5*, *SoNHX11*, *SoNHX12*, *SoNHX13*, *SoNHX14*, *SoNHX26*, *SoNHX33*, and *SoNHX42*. Similarly, ath-miR398 was found to target eight genes (*SoNHX6*, *SoNHX13*, *SoNHX14*, *SoNHX18*, *SoNHX19*, *SoNHX20*, *SoNHX26*, and *SoNHX27*), making it another prominent miRNA in this context. In contrast, ath-miR161, ath-miR162, ath-miR164, ath-miR165, and ath-miR396 each targeted only a single gene, specifically *SoNHX36*, *SoNHX30*, *SoNHX10*, *SoNHX41*, and *SoNHX29*, respectively. Furthermore, several *SoNHX* genes were targeted by multiple ath-miRNAs. For example, *SoNHX9* was targeted by ath-miR167, ath-miR169, ath-miR171 and ath-miR390; *SoNHX11* by ath-miR156, ath-miR157 and ath-miR167; *SoNHX13* by ath-miR156, ath-miR170, ath-miR390 and ath-miR398; *SoNHX14* by ath-miR156, ath-miR170, ath-miR390 and ath-miR398; *SoNHX16* by ath-miR167, ath-miR169 and ath-miR393; *SoNHX27* by ath-miR157, ath-miR169, ath-miR390 and ath-miR398; *SoNHX30* by ath-miR159, ath-miR162 and ath-miR172; *SoNHX36* by ath-miR157, ath-miR161, ath-miR171 and ath-miR397; *SoNHX42* by ath-miR156, ath-miR171 and ath-miR397; and *SoNHX43* by ath-miR159, ath-miR319, ath-miR395 and ath-miR397 (Figure 9). This comprehensive analysis suggests that *SoNHX* genes interact with numerous stress-related miRNAs, and that certain genes are particularly prominent as frequent miRNA targets, potentially reflecting their involvement in stress response regulation.

## 4. Discussion

In this study, a comprehensive genome-wide analysis of the *NHX* gene family in *Spinacia oleracea* was conducted; gene structures, protein-level characteristics, phylogenetic relationships, and regulatory elements were examined in a multifaceted manner. The identification of 44 *SoNHX* genes distributed across all six chromosomes reveals that this gene family is extensively represented in the spinach genome and may play roles in a wide variety of cellular functions. The notably higher density of *NHX* genes on chromosome 6 of the *S. oleracea* suggests that this region has played a significant role in the evolutionary expansion of the *NHX* gene family. This gene clustering suggests the occurrence of tandem or segmental duplications on chromosome 6, shaping a genomic landscape conducive to the diversification and functional specialization of *NHX* genes. Additionally, the co-localization of these genes may facilitate coordinated gene expression, potentially leading to critical effects in physiological adaptations of the plant, such as salt stress tolerance, pH balance, and ion homeostasis. Furthermore, the high number of genes on chromosome 4 supports the important role of this chromosome in the functional adaptation of the gene family. On the other hand, the low number of *NHX* genes on chromosome 2 suggests that this region may have a more limited role in gene family members or that fewer duplications have occurred due to evolutionary constraints. The fact that some gene pairs share exactly the same start positions on the chromosome indicates that these genes could be recent products of tandem duplications. Such gene duplications are critical mechanisms for the functional expansion of the gene family and diversification of adaptive responses [48]. Therefore, the chromosomal organization and structure of these genes provide important insights into the evolutionary dynamics of the *NHX* gene family and the plant’s adaptation to environmental stresses.

Previous studies on *NHX* genes have reported that *Actinidia chinensis* contains 8 [14], *Phaseolus vulgaris* 9 [9], *Morus atropurpurea* 7 [8], *Punica granatum* 10 [26], *Solanum lycopersicum* 7 [25], *Cucurbita moschata* 9, *C. maxima* 9, *C. pepo* 8 [23], *Triticum aestivum* 30 [19], *Beta vulgaris* 5 [24], *Gossypium barbadense* 25 [22], *Canavalia rosea* 8 [21], *Chenopodium quinoa* 10 [7], *Oryza sativa* 7 [17], *Vigna mungo* 44 [12], *Capsicum annuum* 9 [10], *Amaranthus tricolor* 9 [20], *Medicago truncatula* 8 [15], and *Solanum tuberosum* 25 *NHX* genes [13]. In the present study, a total of 44 *NHX* genes were identified in *S. oleracea*, indicating that this species harbors a greater number of *NHX* genes compared to many other plant species. This elevated gene count may suggest a potential involvement of *NHX* genes in plant responses to diverse environmental stresses. However, further experimental studies will be required to confirm their specific roles in stress tolerance. Moreover, the genetic diversity within this family may allow distinct NHX members to perform specialized roles in different tissues or developmental stages, potentially contributing to the physiological flexibility of the plant.

To provide a more detailed understanding of the evolutionary relationships within the *NHX* gene family and the structural diversity among the genes, a phylogenetic analysis was conducted. The findings clearly revealed a distinct evolutionary divergence among the SoNHX proteins. In particular, the dense branching pattern observed in Group III suggests that the genes within this group possess a high degree of genetic variability and may have evolved to perform diversified biological functions. This indicates a functional specialization that likely developed over the course of evolution. Furthermore, the distinct separation of genes such as *SoNHX1*, *SoNHX9,* and *SoNHX33* in the phylogenetic tree implies that these genes may have acquired unique functions and diverged evolutionarily in different directions.

Structural modeling data also provided findings that support the results of the phylogenetic analysis. In particular, the SoNHX9 protein was notable for containing a high proportion of disordered regions, a feature that corresponded with its phylogenetically isolated position. The abundance of disordered regions suggests that this protein may possess a high interaction capacity and a structurally flexible conformation responsive to environmental changes. Similarly, the proportions of α-helices, β-strands, transmembrane helices (TM helices), and disordered regions in SoNHX18, SoNHX19, and SoNHX20 proteins showed a high degree of similarity, implying that these proteins may have originated from a common ancestor and are likely to share similar biological functions. The grouping of these three proteins within the same clade in the phylogenetic tree further reinforces this evolutionary proximity. Analysis of Group II members revealed that the proteins in this group largely exhibit comparable structural features. Notably, β-strand structures were either completely absent or present at very low levels (1–4%) in most proteins of this group. The complete absence of β-strand structures in proteins such as SoNHX10, SoNHX16, SoNHX17, SoNHX18, SoNHX19, SoNHX20, and SoNHX41 suggests that they share similar folding patterns and likely perform common or closely related biological functions. These shared structural characteristics may enhance the proteins’ responsiveness to environmental stress by enabling the conformational flexibility necessary for efficient ion exchange and signal transduction.

Homology modeling results revealed that NHX proteins exhibit considerable structural diversity at the tertiary structure level. Notably, proteins such as SoNHX13, SoNHX14, and SoNHX39, characterized by the presence of multiple structural domains comprising both α-helices and β-sheets, are hypothesized to serve pivotal functions in cellular signal transduction pathways. Their complex architecture, including extended loop regions, is likely to facilitate the formation of transient protein–protein interactions, thereby enabling these proteins to engage dynamically in various regulatory processes. These structural attributes imply that NHX proteins may function beyond their canonical roles in ion homeostasis and could be actively involved in modulating intracellular signaling networks. This multifunctional potential indicates that these proteins may contribute to plant responses to environmental stress by participating in regulatory networks involved in stress perception and adaptation.

Conserved motif analysis further corroborates the observed structural similarities among these proteins. Notably, SoNHX16 and SoNHX17 contain only the ninth motif, while SoNHX10, SoNHX18, SoNHX19, SoNHX20, and SoNHX41 possess merely two motifs. This pattern suggests that these proteins are evolutionarily conserved yet exhibit limited functional diversity. Their clustering within the same branch of the phylogenetic tree serves as strong evidence of their structural and evolutionary relatedness. Furthermore, the repeated occurrence of Motif 9 across multiple proteins underscores its evolutionary conservation within the NHX family and implies a fundamental biological role. Another notable observation is the presence of the tenth motif in SoNHX26, SoNHX28, SoNHX30, and SoNHX33, as well as the occurrence of two copies of the fifth motif in SoNHX6, SoNHX8, SoNHX21, SoNHX22, and SoNHX23. These motif variations suggest potential functional specialization or enhanced regulatory capacity in these proteins. Such variations may reflect adaptive evolutionary modifications that allow these proteins to fulfill distinct roles or respond more effectively to environmental stimuli. Together, these differences in motif composition likely contribute to the functional diversification of NHX proteins, enabling specific members to play specialized roles in plant stress adaptation and cellular homeostasis maintenance.

Gene structure analysis strongly supports the molecular basis of the phylogenetic distinctions. The notable differences in exon–intron organization reveal that *SoNHX* genes have undergone extensive genomic rearrangements throughout their evolutionary history. For instance, genes such as *SoNHX13* and *SoNHX14*, which possess a high number of exons and introns, are likely controlled by complex regulatory mechanisms and perform potentially multilayered functions. In contrast, genes with simpler structures, such as *SoNHX2*, which contains only two exons and one intron, may be associated with distinct expression dynamics and specialized functional roles. Notably, genes phylogenetically clustered in Group III exhibit a more compact gene structure, characterized by 2–4 exons and 1–3 introns, compared to genes in other groups. This feature implies that Group III genes exhibit a more rapid and adaptable regulatory capacity, facilitating dynamic responses to environmental stimuli or cellular requirements. Likewise, the variation in exon/intron organization among different *NHX* gene groups in *Phaseolus vulgaris* suggests that structural modifications—such as exon or intron gain and loss—have contributed to the evolutionary diversification and expansion of this gene family in the species [9]. Similarly, in *Triticum aestivum*, it has been reported that [19] genes within the same group display highly similar intron and exon distributions, with exon lengths and intron regions of genes belonging to the same class being largely conserved. These structural variations in gene organization likely contribute to the differential regulation and functional diversification of *NHX* genes, enabling plants to optimize their responses to environmental stresses and maintain cellular homeostasis.

Protein–protein interaction analyses reveal that the SoNHX protein family forms a dense and complex interaction network within the cell. Examination of this network indicates that SoNHX14, SoNHX20, and SoNHX33 exhibit relatively high connectivity within the predicted interaction network, suggesting that these proteins may occupy central positions in the network and could be involved in coordinated cellular processes. Their relatively central positions in the predicted network may indicate potential involvement in physiological processes such as ion homeostasis, pH regulation, and abiotic stress responses. Conversely, proteins with fewer interactions, including SoNHX38 and SoNHX40, are situated in the peripheral regions of the network, which may indicate functional specialization or a narrower spectrum of activities. The presence of both direct and indirect interactions throughout the network suggests that SoNHX proteins operate within a modular framework, functioning in an integrated and coordinated manner. These findings suggest that NHX proteins are involved not only in ion transport but also play coordinated roles in broader biological processes, including signal transduction, protein stability, and stress tolerance. These results are supported by previous studies in *Morus atropurpurea* [8], where the *NHX* gene family was similarly found to be divided into three distinct subgroups, and *MaNHX* members exhibited diverse and complex expression patterns in response to various signaling molecules, phytohormones, and abiotic stress conditions. Additionally, protein–protein interaction analyses in *Punica granatum* have indicated that PgNHX proteins play important roles in salt stress resistance [26]. Overall, the interaction patterns highlight the central importance of SoNHX proteins in regulating multifaceted physiological pathways, which contribute to enhanced stress tolerance and adaptive capacity in plants.

Subcellular localization analyses provide important insights into the functional diversity and spatial distribution of the SoNHX protein family. Predicted subcellular localization results suggest that SoNHX proteins are mainly associated with membrane-related cellular compartments, including the plasma membrane. Notably, SoNHX34 was predicted to localize exclusively to the peroxisome, SoNHX13 to the nucleus, and SoNHX37 primarily to the plasma membrane, suggesting that these members may perform unique cell type or signal-specific functions. Furthermore, the predicted presence of SoNHX proteins in organelles such as the endoplasmic reticulum (32 proteins) and vacuole (31 proteins) suggests their potential involvement in diverse cellular processes, including ion storage, protein folding, trafficking, and homeostatic regulation. Collectively, these findings suggest that the SoNHX family may constitute a structurally and functionally multilayered and regionally specialized network. Highly connected proteins situated at the core of the interaction network, particularly those associated with membrane systems such as the plasma membrane and endoplasmic reticulum, may facilitate responses to extracellular signals. In contrast, proteins occupying peripheral positions with limited interactions may perform more specialized roles within distinct cellular organelles. Overall, these results suggest that SoNHX proteins could contribute to the regulation of intracellular homeostasis and potentially participate in plant adaptive responses to environmental stress. In contrast to the current study, a previous analysis in *Punica granatum* [26] reported that most NHX proteins were predicted to be localized to the vacuoles, with fewer proteins associated with the plasma membrane. Similarly, a study conducted in *Glycine max* [16] suggested that most GmNHX proteins are localized to the vacuolar membrane. Altogether, the diverse predicted localization patterns of SoNHX proteins in *S. oleracea* indicate their potential involvement in maintaining cellular homeostasis and participating in plant responses to environmental stresses, reflecting complex regulatory mechanisms that may contribute to stress adaptation in spinach.

Cis-acting elements are important sequences located in gene promoters that play a critical role in transcriptional regulation and serve as key factors in controlling the activation and repression of gene expression [49]. A comprehensive analysis of the promoter regions of the *SoNHX* genes provided strong evidence that this gene family is involved in diverse physiological and stress-related processes. Notably, the high number of predicted cis elements identified in the *SoNHX16* gene (144) may suggest potential regulatory complexity. Similarly, *SoNHX32* (142 elements) and *SoNHX33* (135 elements) also exhibited high motif counts, suggesting that these genes may potentially respond to various environmental signals. Furthermore, the identification of stress-related motifs such as LTR, MBS, TC-rich repeats, and WUN-motif in numerous *SoNHX* genes supports their potential roles in environmental stress responses. The presence of such motifs aligns with previous literature findings reporting that the *NHX* gene family plays critical roles in stress tolerance mechanisms and adaptation to conditions such as salinity, drought, and temperature in various plant species [7,12,13,18,22,23,26,50,51]. Additionally, the identification of numerous light-responsive motifs (e.g., G-box, GT1-motif, Box 4, I-box) suggests that these genes may be regulated in processes related to chloroplast development, photomorphogenesis and photosynthesis.

In a comprehensive assessment of cis-acting element motif diversity, the identification of 23 distinct motifs in the *SoNHX21* gene strongly indicates that this gene is intricately regulated by multiple signals. Similarly, the high motif diversity observed in *SoNHX7*, *SoNHX8,* and *SoNHX13* (each containing 22 different motifs) supports the idea that these genes may serve as coordinated targets of various transcription factors and are subject to multilayered transcriptional regulation. In contrast, the *SoNHX30* gene, which contains only eight different motifs, appears to have a relatively limited and specialized regulatory profile and is likely activated under specific physiological conditions. On the other hand, the *SoNHX9* gene, which exhibits the lowest total number of cis-acting elements (69), is likely regulated by a simpler yet fundamentally essential regulatory mechanism. Phylogenetic analyses reveal that this gene is distinctly separated from others, while its protein product shows a unique localization pattern restricted to the Golgi apparatus, vacuole, and plasma membrane. Structurally, compared to other family members, SoNHX9 contains a higher proportion of disordered regions and fewer transmembrane helices, suggesting a distinctive structural conformation. Genomically, the gene has fewer exons and introns, and miRNA target analysis ranks it third in terms of the number of targeting miRNAs. This unique combination of features strongly suggests that *SoNHX9* possesses a specialized and complex post-transcriptional regulatory profile and likely fulfills a critical and specific biological function distinct from other members of the gene family.

A significant finding is the widespread presence of motifs associated with hormonal regulation within the *SoNHX* genes. The extensive distribution of cis-elements such as ABRE (abscisic acid), TGA-element (auxin), CGTCA-motif and TGACG-motif (methyl jasmonate), GARE-motif and P-box (gibberellin), as well as TCA-element and SARE (salicylic acid), suggests that these genes actively participate in phytohormone-mediated growth, development, and stress response pathways. Notably, the pronounced enrichment of ABRE in *SoNHX16*, G-box in *SoNHX32*, ARE in *SoNHX38*, and TC-rich repeats in *SoNHX33* indicates a heightened sensitivity of these genes to their respective hormonal signals. Furthermore, the exclusive occurrence of certain motifs in specific genes is particularly striking: 4cl-CMA2b exclusively in *SoNHX28*, ACA-motif in *SoNHX14*, chs-CMA2a in *SoNHX38*, Gap-box in *SoNHX1*, SARE in *SoNHX32*, and TGA-box in *SoNHX26*. The presence of these unique motifs implies that these genes may be activated only in specific tissues, developmental stages, or environmental conditions, reflecting potential functional specialization within the gene family. Collectively, the cis-regulatory structure of the *SoNHX* gene family strongly suggests that these genes have undergone evolutionary adaptation to effectively respond to a wide range of physiological conditions, including environmental stresses, hormonal signaling, and developmental processes. These findings provide a strong foundation for future investigations, including comprehensive gene expression profiling and further functional characterization studies. The conducted miRNA target analyses already offer valuable insights into the post-transcriptional regulation of this gene family. Such approaches will be important for better understanding the precise biological roles and complex regulatory mechanisms of these genes.

miRNAs are RNA molecules that are 21–23 nucleotides in length and play roles in hormone signaling, plant development, and stress responses [52]. The miRNA interaction analysis demonstrated that the *SoNHX* gene family is extensively regulated at the post-transcriptional level by numerous stress-related *Arabidopsis thaliana* miRNAs. Notably, *SoNHX11*, *SoNHX33*, and *SoNHX9* were predicted to have a relatively large number of potential miRNA-binding interactions, suggesting possible post-transcriptional regulatory associations that require experimental validation. In contrast, *SoNHX18*, which is targeted by only 98 miRNAs, appears to be more specifically regulated, potentially reflecting its role in limited or condition-specific responses. Among the identified miRNAs, ath-miR156 exhibited the highest targeting frequency across the *SoNHX* genes, followed by ath-miR398, indicating that these miRNAs may function as central regulators within this gene family. In contrast, miRNAs such as ath-miR161, ath-miR162, ath-miR164, ath-miR165, and ath-miR396, which each target only a single gene, are likely involved in more selective and gene-specific regulatory mechanisms. Moreover, several genes including *SoNHX9*, *SoNHX13*, *SoNHX14*, *SoNHX27,* and *SoNHX36* are targeted by multiple stress-associated miRNAs, which may point to their participation in complex and overlapping abiotic stress response pathways. Considering that many of these miRNAs have previously been reported to function in abiotic stress regulation [53,54,55,56,57,58,59,60], the results strongly suggest that *SoNHX* genes may be regulated through these miRNAs under environmental stress conditions.

Previous studies have further elucidated the specific roles of certain miRNAs in response to diverse abiotic stress conditions across different plant species. For instance, miR393 has been reported to respond to salt and osmotic stresses in *Arabidopsis thaliana*, salt, alkali, and drought stresses in *Oryza sativa*, cold stress in *Panicum virgatum* L., and waterlogging stress in *Zea mays* L. [61]. miR169 has been reported to be downregulated under drought stress in *Arabidopsis* but upregulated in *O. sativa*; in *Gossypium hirsutum* L., miR156, miR157, miR162, miR172, miR397, miR398, and miR399 were found to be downregulated in both leaf and root tissues under different salt concentrations [56]. miR396 has been associated with enhanced drought tolerance in *Arabidopsis*, and its expression was found to be downregulated in *Oryza sativa* and *Vitis vinifera* L. under drought conditions [55]. Furthermore, miR156 has been implicated in cold tolerance in *Solanum lycopersicum*, while miR167, miR169, miR172, and miR393 were also responsive to cold stress, with miR172 showing the highest expression levels [60]. Other studies have indicated that miR156, miR166, miR319, and miR408 contribute to both biotic and abiotic stress resistance, with miR156 playing a key role in drought response in *Camellia sinensis* (L.) Kuntze [62]. The role of miR393 in drought stress has also been reported in *Arabidopsis*, *Medicago*, *Phaseolus*, and *Oryza* species [63]. Collectively, these findings suggest that the *SoNHX* genes targeted by such miRNAs may potentially be involved in stress-related signaling pathways and could play roles in plant adaptive responses. The consistency of these findings across diverse plant species suggests that various miRNAs may play important roles in mediating abiotic stress responses. These findings reinforce the hypothesis that the *SoNHX* genes, as potential targets of these miRNAs, may be involved in intricate and overlapping signaling pathways associated with stress adaptation. In this context, the application of a bioinformatic approach allowed for a systematic and comprehensive exploration of putative miRNA–gene regulatory interactions. Although experimental validation was not within the scope of this study, the predictions were based on reliable databases supported by peer-reviewed studies and widely used in the scientific community, thereby providing credible and biologically meaningful preliminary insights. Overall, the results not only align with previously documented stress-responsive miRNA behavior but also serve as a valuable foundation for future experimental validation and functional characterization.

## 5. Conclusions

This study presents a comprehensive analysis of the evolutionary, structural, and functional characteristics of the *NHX* gene family in *Spinacia oleracea*, revealing substantial structural diversity and complex regulatory networks within the family. The identification of 44 *SoNHX* genes, notably clustered on chromosomes 4 and 6, provides strong genomic evidence for the evolutionary expansion and functional diversification of this gene family. Phylogenetic, conserved motif, and protein–protein interaction analyses demonstrate that SoNHX proteins exhibit both conserved features and unique functional divergences. Their diverse subcellular localizations and extensive interaction networks further underscore their central roles in ion homeostasis, signal transduction, and stress response pathways. Analyses of promoter cis-elements and miRNA target predictions reveal that the *SoNHX* gene family is subject to complex transcriptional and post-transcriptional regulation, facilitating rapid and precise adaptation to abiotic stresses. Particularly, genes such as *SoNHX9*, *SoNHX16*, and *SoNHX33* hold pivotal positions within this complex regulatory network, contributing significantly to stress tolerance. These findings advance our understanding of the molecular evolution and functional diversity of the *NHX* gene family, while identifying promising targets for genetic and biotechnological strategies aimed at improving abiotic stress resistance in spinach. Future studies may focus on experimental validation of selected *SoNHX* genes through gene expression analyses under abiotic stress conditions and functional characterization using gene overexpression or gene-silencing approaches. Such studies will help clarify the precise roles of these genes in plant stress adaptation and support the development of innovative strategies for improving abiotic stress tolerance in spinach.

## Data Availability

The datasets generated during and/or analysed during the current study are available from the corresponding author on reasonable request.

## Supplementary Materials

The following supporting information can be downloaded at https://www.mdpi.com/article/10.3390/genes17030329/s1: Table S1: Physicochemical properties of SoNHX proteins identified in *Spinacia oleracea*; Table S2: Predicted cis-acting regulatory elements in the promoter regions of *SoNHX* genes; Table S3: Predicted miRNA target interactions for *SoNHX* genes.
